# General practice utilisation by Australian cancer patients in the last year of life

**DOI:** 10.1093/fampra/cmae062

**Published:** 2024-11-12

**Authors:** Matthew P Grant, Damien McCarthy, Chris Kearney, Anna Collins, Vijaya Sundararajan, Joel J Rhee, Jennifer A M Philip, Jon D Emery

**Affiliations:** Palliative Nexus Research Group, Department of Medicine, University of Melbourne, 41 Victoria Parade, Fitzroy, 3065, Melbourne, Australia; Department of Palliative Medicine, St Vincent’s Hospital Melbourne, 41 Victoria Parade, Fitzroy, 3065, Melbourne, Australia; Centre of Expertise in Palliative Care Utrecht, Department of General Practice, Julius Centre, UMC Utrecht, Universiteitsweg 100, 3584CG Utrecht, The Netherlands; Department of General Practice and Primary Care, Centre for Cancer Research, University of Melbourne, 780 Elizabeth St, Melbourne VIC 3010, Australia; Department of General Practice and Primary Care, Centre for Cancer Research, University of Melbourne, 780 Elizabeth St, Melbourne VIC 3010, Australia; Palliative Nexus Research Group, Department of Medicine, University of Melbourne, 41 Victoria Parade, Fitzroy, 3065, Melbourne, Australia; Department of Palliative Medicine, St Vincent’s Hospital Melbourne, 41 Victoria Parade, Fitzroy, 3065, Melbourne, Australia; La Trobe University, Public Health, Science Drive, Melbourne, Victoria 3086, Australia; Discipline of General Practice, School of Population Health, Faculty of Medicine and Health, UNSW Sydney, Botany Street, Kensington, NSW 2052, Australia; Palliative Nexus Research Group, Department of Medicine, University of Melbourne, 41 Victoria Parade, Fitzroy, 3065, Melbourne, Australia; Department of Palliative Medicine, St Vincent’s Hospital Melbourne, 41 Victoria Parade, Fitzroy, 3065, Melbourne, Australia; Department of General Practice and Primary Care, Centre for Cancer Research, University of Melbourne, 780 Elizabeth St, Melbourne VIC 3010, Australia

**Keywords:** palliative care, general practice, primary care, terminal care, home visits, cancer

## Abstract

**Objectives:**

General practice plays a key role in end-of-life care, yet the extent of this remains largely unknown due to a lack of detailed clinical data. This study aims to describe the care provided by General Practitioners (GPs) for people with cancer in their last year of life.

**Methods:**

Retrospective cohort study using linked routine primary care and death certificate data in Victoria, Australia. Patients were included who died from cancer between 2008 and 2017.

**Results:**

In total 7025 cancer patients were included, mean age of 74.8 yrs. 95% of patients visited their GP in the last 6 months of life, with a median of 11 general practice contacts in this period. 72% of patients visited their GP in the second-last month prior to death, and 74% in the last month of life. The majority of patients (58%) were prescribed opioids, 19% anticipatory medications, 24% received a home visit, and a small proportion had imaging (6%) in the last month and pathology (6%) in the last fortnight. Patients in regional areas had more contact with general practices in the last year of life compared to metropolitan patients (median metropolitan = 16, inner regional = 25, and outer regional = 23, *P* < .001). The use of GP services did not differ by cancer type.

**Conclusions:**

GP’s play a central role in end-of-life care provision for cancer patients, which intensifies in the last months of life. There is room for improvement, with a proportion having little or no engagement, and low rates of home visits and anticipatory medication prescribing.

**Key messages**

- General practice plays a key role for cancer patients at the end of life.
- Cancer patients had a median of 19 GP contacts in the last year of life.
- Patients in rural areas accessed general practice more frequently.
- Few patients accessed home visits and anticipatory medications.

## Introduction

General practice has a central role in providing care for cancer patients towards the end of life [1–3]. The role is broad, addressing supportive care needs through treatment, collaborating with specialist teams, managing comorbid conditions, addressing the psychosocial and existential concerns of patients and their families, and providing palliative and terminal care in the home setting [1–3]. In the last months of life, the role of general practice takes on greater significance, as patients with cancer may cease disease-modifying treatments and require increasing support in the home setting. Hospital admissions and interventions may be of diminishing or no benefit, or have the potential to cause harm and deprive patients and their families of time spent together at home [4, 5]. International studies have demonstrated that the involvement of general practitioners (GPs) during this period increases the probability of dying at home, decreases hospitalisations and health care expenditure, and reduces aggressive interventions at the end of life [4–7]. With an ageing world population and increasing incidence of cancer, and thus the number of patients who will require palliative care, the role of the general practice is of increasing importance to meet the care needs of this growing population, to support patients, and promote quality of life in the home setting [8].

The final year of a person’s life is the most resource-intensive in terms of health system usage. Patients in the last year of life make up less than 1% of the population, yet meeting their increasing care needs accounts for between 8% and 11% of overall healthcare expenditure [9]. The majority (65%–80%) of this expenditure occurs in hospitals, where a large proportion of people die, some 50% in Australia [7, 10]. In contrast, the cost of general practice services—including out-of-hours contacts and home visits—are responsible for less than 15% of health care expenditure during this period [7]. In England, patients with cancer attend an average of 43 general practice consultations in the last year of life, compared to an average of 20 consultations for all patients in the last year of life [7, 11].

In the Australian context, the exact contribution of general practice in providing care for cancer patients at the end of life is not fully understood. Anecdotally, it is recognised that many patients disengage from their primary care practitioners during acute cancer treatment, and may fail to re-engage further along the disease course, or their care may be primarily managed by specialist palliative care services [12, 13]. While there is considerable research examining hospital service provision, there is little known regarding the associated usage of general practice services by cancer patients. This study aims to describe the use of general practice by Australian cancer patients in their last year of life and explore demographic and clinical characteristics associated with general practice utilisation.

## Methods

### Study design

This study was designed as a retrospective cohort study incorporating routine clinical and administrative data from Australia. It is reported conforming to the Strengthening the Reporting of Observational Studies in Epidemiology (STROBE) Statement [14]. This research was approved by the institutional review boards of St Vincent’s Hospital Melbourne (LLR 074/19) and National Prescribing Service MedicineInsight Data Governance Committee (2020-013).

### Data sources

This study incorporated data from two datasets, linked through Biogrid Australia. The MedicineInsight dataset (previously with National Prescribing Service MedicineWise and now under the custodianship of the Australian Commission on Safety and Quality in Health Care) includes longitudinal data from primary care clinics as part of routine clinical care, incorporating data from approximately 10% of Australian general practice clinics [15]. We utilised only the cohort of the MedicineInsight dataset from Victoria, Australia. The National Death Index (NDI) describes all Australian deaths containing person-level records of all deaths. These datasets include de-identified demographic, clinical, and administrative details, including causes of death, primary care consultations, billing codes, and clinical care processes such as prescribing, imaging, and laboratory investigations.

### Setting and population

The data included patients with cancer who died in Victoria between 1 July 2008 and 31 December 2017. Victoria is a southern state of Australia with a population of almost seven million people. Inclusion criteria included over the age of 18 at death, at least one primary care encounter in the include practices in the 12 months prior to death, and, who died from a cause related to cancer as listed on the death certificate.

General practice in Australia operates as a gatekeeper system similar to many health systems in Western Europe [16]. General practice is primarily responsible for the general medical care of patients in the community (including nursing homes), and collaborates with other specialists to provide longitudinal care for patients and their families [16]. In urban locations general practice is provided in the community and nursing homes, but in rural areas GPs will often have admitting rights to hospitals and provide in-patient care. Clinical consults are reimbursed by the government, although there may be out-of-pocket fees depending on the practitioner.

### Data

Patients were selected who had ever had a general practice consultation reason involving cancer or palliative care in the MedicineInsight dataset. Patients were selected from the National Death Index who had a registered date of death from 1/07/2008 to 31/12/2017, to allow for 1 year of MedicineInsight data prior to death. Linkage then proceeded between datasets.

Data were collected from the MedicineInsight and National Death Index datasets describing patient demographics (age, gender, site of care). Cancer diagnoses and other major comorbidities were identified based on the International Statistical Classification of Diseases and Related Health Problems (10th edition), Australian Modification codes [17, 18]. Remoteness was coded according to the Australian Statistical Geography Standard (ASGS) edition 3, which classifies remoteness on five levels, from metropolitan to very remote [19]. Victoria contains only a small remote region consisting largely of a national park, and no very remote regions, although compared to much of the rest of the world, these outer regional regions would be considered remote. Previously described indicators of appropriate end-of-life care as described by Earle and de Schreye were collected through standardised clinical procedure codes at different time points, as were available in the data [20, 21]. Appropriate care process indicators relevant to general practice included prescription of opioids, home visits, and prescription of anticipatory medications in the last three months of life, and inappropriate care included imaging (last month of life) and laboratory testing (last two weeks) [6]. These indicators are population level indicators, and do not describe the appropriateness of this care for the individual patient.

Variables describing primary care provision included number and types of contacts, medication prescribed, and requests for laboratory and imaging tests. Contacts were defined as an episode of care provision which involved the GP or nurse, and were not purely administrative. This included contacts that were not billable. At the time of these data, only physical GP contacts were billable, and thus telephone contact with patients, caregivers and specialists were not reimbursed by the health system or health insurance. Home visits were identified through Medicare Benefits Schedule billing codes and free text describing the reason for consultations. Anticipatory medications were defined as the proactive prescribing of injectable medicines that are commonly required to control symptoms in palliative care, detailed in Supplementary File Table A and based on existing literature [22].

### Data analysis

Patient demographics and clinical characteristics were detailed using descriptive statistics. Dates of admissions, consultations, investigations, prescribing, and interventions were all employed to calculate the time between the event and the death of the patient, and grouped in monthly intervals according to their relation to the date of death. Calculations were expressed as means and standard deviations for parametric data, and medians and interquartile ranges for nonparametric distributions. Patient cohorts were distinguished based on the remoteness classification and classification of cancer type for major cancers. Chi-squared testing was employed for comparisons of difference for categorical variables, and Krushall–Wallis test for nonparametric data. A *P*-value of less than .05 was considered statistically significant. Stata 15 (Stata Corp, 2017, College Station, Texas, USA) was employed for data linkage, and IBM SPSS software version 27 (IBM, 2021, Chicago, Illinois, USA) for analysis.

## Results

A total of 116 956 patients were identified in the MedicineInsight dataset with a clinical diagnosis of cancer or palliative care, for whom 20 832 (17.8%) had a linked date of death in the NDI. Of these patients, 10 267 had cancer listed as a cause of death. A further 3242 patients were excluded due to no contacts in the year prior to death, or where data was incomplete in timeframe. A total of 7025 patients were included in the cohort for analysis. Figure A in the Supplementary File describes this process.

### Patient characteristics

In total 3130 patients (45%) were female, with a mean age of 74.8 years (see Table 1). The most common cancer types were lung (18%), genitourinary (16%), upper (14%) and lower (14%) gastrointestinal tract, haematological, (12%) and breast (9%) cancers. These data are described in Table 1.

**Table 1.** Patient demographic and clinical characteristics.

		*N* = 7025 (%)
*Sex*	Female	3130 (45)
*Age*—mean (SD)		74.8 years (13.1)
*Place of residence*	Metropolitan	4098 (58)
	Inner regional	2476 (35)
	Outer regional	451 (6)
*Cancer type*	Lung	1250 (18)
	Genito-urinary	1097 (16)
	Upper gastrointestinal	953 (14)
	Lower gastrointestinal	951 (14)
	Haematological	835 (12)
	Breast	597 (9)
	Skin	301 (4)
	Gynaecological	289 (4)
	Neurological	182 (3)
	Head and neck	161 (2)
	Unknown primary/other	409 (6)

### Utilisation of general practice services

**Figure 1:**
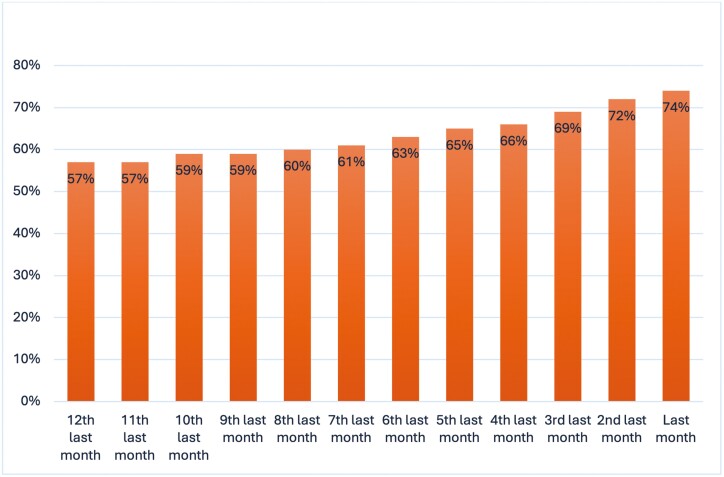
Proportion of patients accessing GP care for each month prior to death.

Table 2 and Figure 1 describe the use of general practice services in the last year of life. Patients had a median of 19 general practice contacts in the last year of life. Most patients had contacts in the last six (95%), three (89%), and one (74%) months of life, as described in Table 2. Figure 1 describes the timing of these contacts, with 57%–61% of patients being involved with their GP between 12 and 7 months prior to death, and this percentage increasing towards the end of life, with 72% of patients accessing GP care in the second-last month prior to death, and 74% in the last month of life.

**Table 2.** Primary care service use in the last year of life.

	Time period	
*GP contacts*	Month 7–12 -med [IQR]	7 [2–13]
	Month 4-6—med [IQR]	4[1–8]
	Last 3 months—med [IQR]	7 [3–12]
	Total contacts last 12 months—med [IQR]	19 [10–31]
*Patients who had contact with GP*	Last 6 months—*n* (%)	6647 (95)
	Last 3 months—*n* (%)	6239 (89)
	Last month—*n* (%)	5205 (74)
*Home visits*	Last 3 months—*n* (%)	1672 (24)
	Last months—*n* (%)	1420 (20)
*Care processes*		
Opioids prescribed	Last 3 months—*n* (%)	4046 (58)
Anticipatory medications	Last 3 months—*n* (%)	1299 (19)
Imaging	Last month—*n* (%)	403 (6)
Pathology	Last 2 weeks—*n* (%)	438 (6)

The majority of patients (58%) were prescribed opioids, 19% were prescribed anticipatory medications, and 24% of patients received a home visit, with 85% of these visits occurring in the last month of life. The proportion of patients who received potentially inappropriate care was low, including imaging (6%) in the last month and pathology testing (6%) in the last fortnight prior to death.

### Remoteness

**Figure 2:**
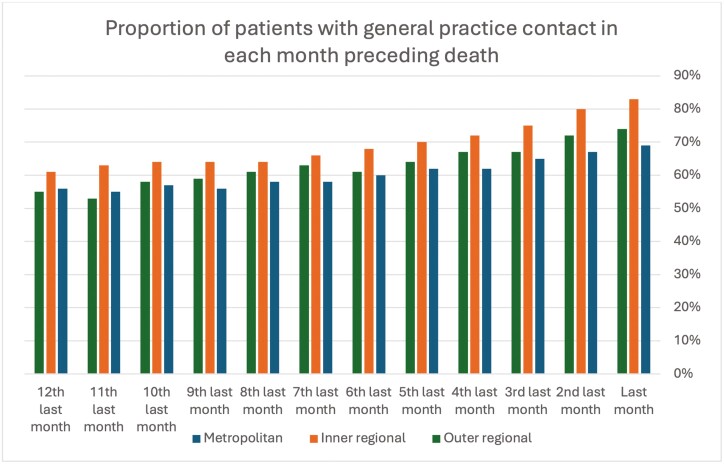
Proportion of patients accessing GP care for each month prior to death according to remoteness.

Patients were grouped into cohorts according to the remoteness classification of general practice site—as metropolitan, inner regional, or outer regional. Patients in regional areas had a greater number of contacts with general practices in the last year of life than those living in metropolitan areas (median contacts metropolitan = 16, inner regional = 25, and outer regional = 23, *P* < .001). The number of contacts was greater in regional patients compared to metropolitan patients across all time periods, but most pronounced in the last three months of life (median contacts metropolitan = 5, inner regional = 10, outer regional = 8, *P* < .001). Table 3 and Figure 2 describe the use of general practice services according to remoteness classification of patients.

**Table 3.** Primary care service use in the last year of life according to remoteness.

	Time period	Metropolitan	Inner regional	Outer regional	*P-value*
*Number patients (%)*		4098 (58)	2476 (35)	451 (6)	
*Age—mean (SD)*		75.3 (13.2)	74.4 (12.7)	72.9 (14.0)	
*GP contacts*	Month 7–12—med [IQR]	6 [2-12]	8 [3-15]	7 [2-14]	*<.001*
	Month 4–6—med [IQR]	4 [1-7]	5 [2-10]	5 [1-10]	*<.001*
	Last 3 months—med [IQR]	5 [2-9]	10 [4-15]	8 [2-14]	*<.001*
	Total contacts last 12 months—med [IQR]	16 [8-27]	25 [14-37]	23 [11-35]	*<.001*
*Care processes*					
Home visits	Last 3 months—*n* (%)	978 (24)	582 (24)	112 (25)	*.821*
Opioids prescribed	Last 3 months—*n* (%)	2166 (53)	1615 (65)	265 (59)	*<.001*
Anticipatory medications	Last 3 months—*n* (%)	809 (20)	417 (17)	73 (16)	*.006*
Imaging	Last month—*n* (%)	140 (3)	223 (9)	40 (9)	*<.001*
Pathology	Last 2 weeks—*n* (%)	143 (4)	262 (11)	33 (7)	*<.001*

Patients in inner and outer regional locations had higher rates of opioid prescriptions than metropolitan patients (65% and 59% respectively, versus 53%, *P* < .001), but received fewer prescriptions for anticipatory medications than patients in metropolitan regions (17% and 16%, versus 20%, *P* = .006). Smaller proportions of metropolitan patients receiving imaging and pathology (3% and 4%) investigations at the end of life, compared to inner regional (9% and 11%) and outer regional (9% and 7%, both *P* < 0.001) patients. Rates of home visits did not differ between the groups.

### Cancer type

Patients were grouped into cohorts according to the classification of cancer type for the major cancer types (lung, genito-urinary, upper gastrointestinal, lower gastrointestinal, haematological, and breast cancers). Table B, Supplementary Files, describes general practice usage according to major cancer types. The number of GP contacts did not differ significantly between cancer cohorts. Care processes such as home visits, anticipatory medications, opioid prescriptions, imaging, and pathology did not demonstrate consistent differences between cancer types.

## Discussion

People with cancer are heavily reliant on GP care in the last year of life, with a median frequency of 19 general practice contacts in this period. This care increases in intensity at the end of life, with more patients accessing GP care in the last two months of life, and an increased quantity of episodes of care, suggesting that this care is responsive to increasing care needs at the end of life. This care is largely appropriate, with few patients receiving inappropriate care processes such as imaging and laboratory testing in the last weeks of life [20, 21]. Whilst many patients received opioid prescriptions, other appropriate care indicators such as home visits or prescribing anticipatory medications were received by only a small proportion of patients. These results highlight the indispensable role of general practice in cancer care, however, there continues to be room for improvement [11].

Cancer patients in regional areas have different patterns of care in the last year of life, having an average of 7–9 more general practice contacts in this period. Victoria is the most densely populated state in Australia, and these differences are likely even further pronounced in more remote regions of Australia. While this is a striking difference, it is not surprising. In Australia and internationally, there are significant disparities in health service access, availability, and affordability in regional and remote areas compared to people living in metropolitan areas, in which general practice may take on a larger role in providing these services [23, 24]. Access to specialist oncology and palliative care services may be limited in regional areas, and thus general practice services are likely accessed in order to meet these care needs, with GPs taking on a broader scope of practice with greater involvement. Lynch and Ding highlighted that these limitations in access for patients in rural areas add to the complexity of their care [25, 26]. Research in palliative care patients in Canada, a country that is also faced with significant geographical limitations as in Australia, identified that rural patients had far higher health care expenditure at the end of life, with increased use of both primary care services and inpatient hospital care [27]. This is consistent with previous research that patients in rural areas are more likely to die in a hospital, which may be related to the limitations of both formal and informal care structures [28]. In general, healthcare outcomes of patients in regional and remote areas are worse than in metropolitan regions, yet this is not evident through examining the quality indicators of end-of-life care in these results [24]. Patients in regional areas were more likely to have opioids prescribed, and similar rates of home visits, yet did receive increased investigations at the end-of-life, however, this may be explained by decreased access of specialist services where these investigations would have otherwise occurred.

The utilisation of general practice services at the end of life varies considerably between countries, which is likely related to the variation in the organisation of health systems, how general practice services operate within these systems, and cultural and economic influences. In the USA, 70%–78% of patients accessed their GP in the last six months of life, with a mean 1.9–2.4 consults in this period [29]. In comparison, 89% of patients in Belgium accessed GP care in the last three months of life, with a median of six visits, and patients dying at home in Belgium and the Netherlands received an average of five GP contacts in the last month of life [11, 30, 31]. In the UK, whose health system bears many similarities to Australia, the majority of patients (87%) access GP care in the last year of life, receiving on average 20–43 GP contacts in this period [7, 11, 32]. These European studies are consistent with our results, and demonstrate the increasing intensity of general practice care utilisation in the last six months of life, peaking in the last two months [7, 11, 30, 32]. It is hypothesised that this increasing intensity is related to the increasing care needs of patients and their families in the last months of life, which is even more pronounced in the cohort of patients who die at home [6, 30]. While these studies describe patterns of utilisation, such large-scale cohort studies are limited in their ability to describe the complexity of this care provision, which increases in these last months of life, as patients and carers have increasing care needs requiring more complicated medical management and care coordination through the general practice team [3, 11].

These results reinforce the essential and central role of general practice for patients with cancer, and it is paramount that health policy continues to support and strengthen this role for the future. The future of health care in developed countries will be defined by an ageing population with an increased prevalence of cancer and organ failure, for whom general practice is ideally placed to provide appropriate and quality care, that is cost-effective, and promotes living and dying in the home environment [9, 33]. However, providing quality palliative care through general practice is not without challenges. It is labour-intensive work, with patients receiving many consultations in the last months of life for complex medical issues, which may be out-of-hours and require extensive collaboration with other health providers [1]. Reimbursement for home visits and providing this complex care required for these patients is limited in Australian general practice [6]. These barriers may be exacerbated by some GPs having limited formal training directed towards managing these complex and multimorbid issues in the home setting [11, 34]. Home visits and prescribing of anticipatory medications are both interventions associated with quality end-of-life care yet occur sparingly in practice [6]. A systematic review by Rhee et al highlighted that international patients in rural locations utilise general practice services differently at the end of life, which is driven by a broad array of patient, health care provider, practice, and system factors [34]. Both Rhee *et al*. and Gao *et al*. highlighted that practitioner factors are a major influence, with younger, urban, part-time, and less experienced GPs being less likely to be involved in end-of-life care for patients, and thus there is a need for education and training to up-skill these practitioners so that they are able to address the care needs of these patients, and for health systems to better support and reimburse general practice to provide this care [11, 34]. Health policy and research should ideally focus on how general practice can be better supported to deliver quality care for patients in the last year of life, and which policy mechanisms may promote this care. There is a small proportion of cancer patients who do not engage with their GP in the last months of life, and it is unclear who these patients are, their specific care needs, or how their needs could potentially be addressed in the general practice setting.

This study incorporates a large cohort of Australian cancer patients utilising linked routine primary care and death index data, and constructs a detailed mapping of how cancer patients access primary care services in the last year of life. Previous studies have largely used billing data, and this study enables a more detailed exploration through the use of general practice clinical data, incorporating information on prescribing, investigations, and diagnostic information. In this cohort, we included patients who died of cancer as listed on their death certificate, and we did not seek to include patients who may have died of a cause unrelated to cancer or with a background cancer diagnosis.

The use of such data has limitations. Primary care electronic records in Australia do not routinely use diagnostic coding such as the ICD-10, and dates of death may not be reliably recorded or missing. The linkage with death index data was necessary to ensure this accuracy, comprising dates and coded diagnostic causes of death. There were numerous variables of interest that were not available in these data, such as place of death and involvement of palliative care services. The general practice data employed comprises approximately 10% of the Victorian population, however as patients are not registered to a single GP, they may visit more than one GP practice in the last year of life. We included only active patients of the practices, however, it is likely that a small number of patients accessed other general practice services during this timeframe (e.g. whilst on holiday or visiting family), and thus the data may be a slight under-representation of general practice utilisation. This data is specific to the use of general practice services in Victoria, Australia, and thus these results may not be readily generalisable to other contexts due to the substantial variability in health service access and organisation internationally.

## Conclusions

These data make evident the central role of general practice in providing care for people with cancer in the last year of life. It is care that is largely appropriate, accessible, and, in regional areas, is of even greater importance to provide much-needed care at the end of life. Despite this, there is room for improvement, particularly to facilitate access to home visits and anticipatory medications, and promote access for the small proportion of cancer patients who are not engaged with their GP in the last months of life.

## Supplementary Material

cmae062_suppl_Supplementary_File

## Data sharing statement

This work has utilised routine clinical and administrative data on an individual level, containing information that could compromise the privacy of participants. The data are thus not publicly available. The data may be made available upon reasonable request, depending on their use, with enquiries directed towards the corresponding author (M Grant).
