# Supplementary material for: General practice utilisation by Australian cancer patients in the last year of life
Source: Fam Pract. 2024 Nov 12;42(2):cmae062. doi: 10.1093/fampra/cmae062 (PMC11809245; doi:10.1093/fampra/cmae062)
Supplement: cmae062_suppl_Supplementary_File [file cmae062_suppl_supplementary_file.pdf]

Supplementary files

Active medication	Form
Morphine	ampule for injection
Fentanyl	ampule for injection
Oxycodone	ampule for injection
Hydromorphone	ampule for injection
Metoclopramide	ampule for injection
Cyclizine	ampule for injection
Haloperidol	ampule for injection
Midazolam	ampule for injection
Clonazepam	ampule for injection
Glycopyrrolate	ampule for injection

Table A – Anticipatory medications

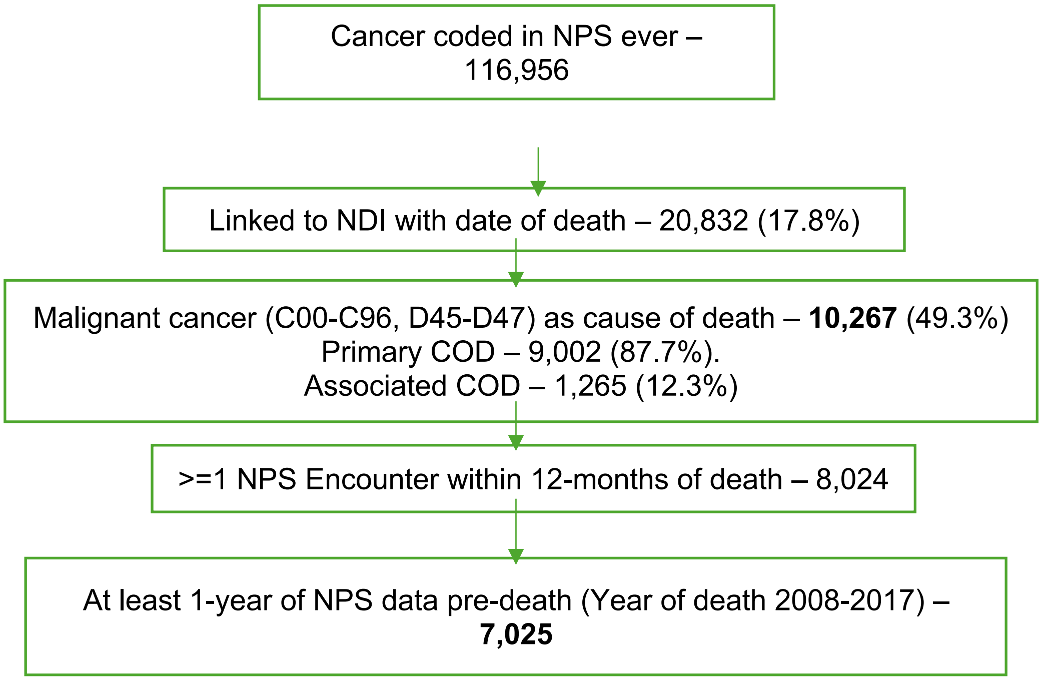

Figure A – Flow diagram describing patient inclusion.

	Time period	Lung	GU	Upper GI	Lower GI	Haem	Breast
**Number patient** (%)		1250 (18)	1097 (16)	953 (14)	951 (14)	835 (12)	597 (8)
**Age** – mean (SD)		73.8 (10.6)	80.1 (10.5)	72.8 (12.5)	75.6 (13.1)	77.1 (12.6)	72.0 (16.0)
**GP contacts**	Month 7-12 -med [IQR]	7 [2-12]	9 [4-15]	6 [2-12]	7 [2-13]	7 [3-13]	6 [2-12]
	Month 4-6 – med [IQR]	4 [2-8]	5 [2-9]	4 [1-8]	4 [1-8]	4 [1-8]	3 [1-7]
	Last 3 months – med [IQR]	7 [3-12]	8 [3-12]	7 [3-12]	6 [3-12]	5 [2-11]	5 [1-11]
	Total contacts last 12 months – med [IQR]	19 [10-30.25]	22 [12-33]	19 [10.5-30]	18 [9-30]	18 [9-31]	15 [7-28]
**Care processes**							
Home visits	Last 3 months – n (%)	279 (22)	316 (29)	181 (19)	237 (25)	173 (21)	150 (25)
Opioids prescribed	Last 3 months – n (%)	766 (61)	659 (60)	601 (63)	569 (60)	351 (42)	306 (51)
Anticipatory medications	Last 3 months – n (%)	211 (17)	196 (18)	199 (21)	191 (20)	108 (13)	108 (18)
Imaging	Last month – n (%)	76 (6)	80 (7)	57 (6)	42 (5)	41 (5)	27 (5)
Pathology	Last 2 weeks – n (%)	65 (5)	93 (9)	53 (6)	53 (6)	73 (9)	22 (4)

Table B – Primary care service use in the last year of life according to major cancer types
GU (Genito-urinary), GI (Gastrointestinal), Haem (Haematology)
*statistical testing of differences was conducted and demonstrated no differences, thus is not presented.
